# Augmented Tendon Repair with Internal Bracing: Surgical Technique

**DOI:** 10.3390/jcm14227963

**Published:** 2025-11-10

**Authors:** Nina Handzewniak, Richard Pearse, Abbie Randall, Abid Mahmood, Tanvir Khan, Shahnawaz Khan, Henry Atkinson

**Affiliations:** 1 Royal Free London NHS Foundation Trust, Pond Street, London NW3 2QG, UK; 2 Royal National Orthopaedic Hospital NHS Trust, Brockley Hill, Stanmore HA7 4LP, UK; 3 Maidstone and Tunbridge Wells NHS Trust, Tonbridge Road, Royal Tunbridge Wells TN2 4QJ, UK

**Keywords:** internal bracing, tendon repair, quadriceps tendon, patellar tendon, Achilles tendon, tendon injury

## Abstract

**Objectives**: The role of internal bracing in lower limb tendon repairs and reconstructions is not widely published. We report our techniques for internal brace-augmented tendon repair (IBA-TR) in the surgical treatment of acute ruptures of the patellar tendon, the quadriceps tendon and the Achilles tendon. **Methods**: The outcomes of 100 cases of IBA-TR treated by a single surgeon over an 8-year period (2014–2022) were retrospectively analysed with a minimum follow-up of 6 months. **Results**: The mean time to mobilisation without a brace was 32 days. Three cases of infection were reported and treated with antibiotics, with no cases of deep infection requiring return to theatre. There were no cases of venous thromboembolism. No cases of failure of tendon repair were observed. Of the 100 patients, 98 returned to their pre-injury level of activity. **Conclusions**: This study represents the largest cohort of tendon repairs augmented with an internal brace to date. With no reported failures or returns to theatre, this repair technique has been demonstrated to be safe and clinically effective, dispensing with the need for cast immobilisation. Level of evidence: III.

## 1. Introduction

Surgical repair is the mainstay of treatment for large tendon ruptures of the lower limb, particularly those relating to the patellar tendon (PT) and quadriceps tendon (QT) [1,2]. Despite surgical intervention, up to fifty percent of patients demonstrate persistent weakness [3]. Furthermore, non-operative management for quadriceps and patellar tendon ruptures is generally reserved for those unfit for surgery. There remains considerable debate surrounding operative vs. non-operative measures for Achilles tendon (TA) ruptures [4,5].

Ligament reconstruction in the lower limb can be augmented with an internal brace (IB), commonly using suture tape. There is in vitro evidence that IB-augmented constructs can withstand a higher load to failure in comparison to conventional repairs [6]. In vitro studies have also demonstrated that mechanical loading is an essential component of tendon healing, whereas disuse atrophy compromises the extracellular matrix and the tensile strength of the tissue [7]. Mechanically enhancing the repair to permit early protected physiological loading is therefore one of the key goals of surgical intervention while avoiding many of the problems arising from prolonged immobilisation which accompany such injuries [8]. This “mechanical optimisation” of the biological environment coupled with the potential for earlier rehabilitation has led to the increased use of IB augmentation for ankle ligament repairs and reconstructions [9].

The role of internal braces in the repair of acute lower limb tendon ruptures is yet to be widely reported. Isolated case reports have demonstrated their utility and potential for augmenting traditional repairs, although there exists variance in surgical techniques related to internal bracing [10]. A systematic review of 347 patients from nine studies found a reduction in failure rates (10.4%) and favourable PROMs in internally braced anterior cruciate ligament (ACL) repairs [11]. The evidence for internally bracing quadriceps and patella tendon repairs is limited to case reports [12,13,14]. Although not internally braced as described in this article, augmented Achilles tendon repairs in 83 patients from four studies were found in a review to have similar rates of re-rupture when compared to those that were not augmented (7.2% vs. 9.3%, *p* = 0.69), with no significant difference found in complications including infection [15].

In this article we describe techniques for internal brace-augmented tendon repair (IBA-TR) in the surgical treatment of acute ruptures of the patellar tendon, the quadriceps tendon and the Achilles tendons. We also report the clinical outcomes for patients who underwent IBA-TR.

## 2. Methods

All patients were consented to medical photography. This was a single-surgeon retrospective study, thus nominally falling within the remit of service evaluation. Such studies are not required by the UK Integrated Research Application System (IRAS) to submit for ethical approval. All patient data was blinded from the end users. No AI tools were used during the study design, data analysis, or manuscript preparation.

All patients undergoing acute tendon repair for the patellar, quadriceps and Achillies tendons were included, with data prospectively recorded and retrospectively analysed. Chronic tears, concomitant ligament injuries and bony traumas were excluded from the study. The diagnosis was confirmed by clinical examination and formal MR imaging of all patients to exclude concomitant injuries. TA patients received USS scans instead of MR imaging.

Patients were seen at 2, 6, 12 and 26 weeks post-operatively, with targeted physiotherapy throughout the first six months. Four patients were lost to follow up. Tegner scores were routinely collected pre-operatively and then again at 6 months post-surgery. This is a surgical technique paper; thus, no power calculation was performed.

### 2.1. Surgical Techniques for Specific Tendons

Each patient with Achille’s, quadriceps or patellar tendon rupture first underwent a primary repair, which was then augmented with a FiberTape© (Arthrex Inc., Naples, FL, USA) internal brace. The exact surgical technique for each tendon is described below.

### 2.2. IBA-TR of the Achilles Tendon: Surgical Technique

A lateral para-midline incision is made over the tendon at the level of the rupture. Remnants of the paratenon layer are preserved, where practical, in order to suture this over the tendon after repair. Minor adhesions and fibrotic material are resected, and the tendon is debrided back to healthy edges (usually in the more chronic ruptures). Non-absorbable sutures, 2 polyester or SutureTape is used in a Krackow configuration in both the proximal and distal tendon stumps. The stump ends are brought together, and the suture ends are tied to restore the original tendon’s length and tension. One should compare the resting foot position of the repaired side to the contralateral uninjured side to ensure symmetrical restoration. The knotted suture ends are placed at the edge of the repair.

An absorbable 2/O polyglactin 910 epitendinous stitch is used to complete the repair, bringing the frayed tendon strands together. The paratenon fragments are sutured across the top of the repair site with an absorbable suture (2–0 polyglactin 910).

**Figure 1:**
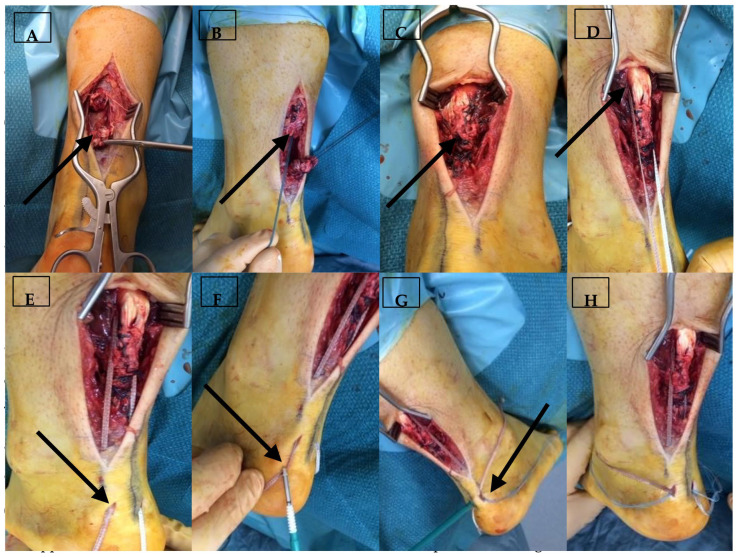
The step sequence for Achilles tendon repair with internal brace implantation. The black arrows point directly to the step of procedure described with each letter. (**A**) ruptured ends of the tendon; (**B**) Krackow sutures on both ends of the rupture; (**C**) direct repair of the Achilles tendon rupture; (**D**) FiberTape© being secured to the proximal part of the healthy tendon/muscle; (**E**) FiberTape© tunnelled to distal stab incisions; (**F**,**G**) FiberTape© being secured distally both medially and laterally into the calcaneum with SwiveLock screws; (**H**) intra-op tensioning of the IBA-TR in maximal ankle dorsiflexion matching the contralateral side, demonstrating where the FiberTape© protects the repair akin to a “seatbelt”.

**Figure 2:**
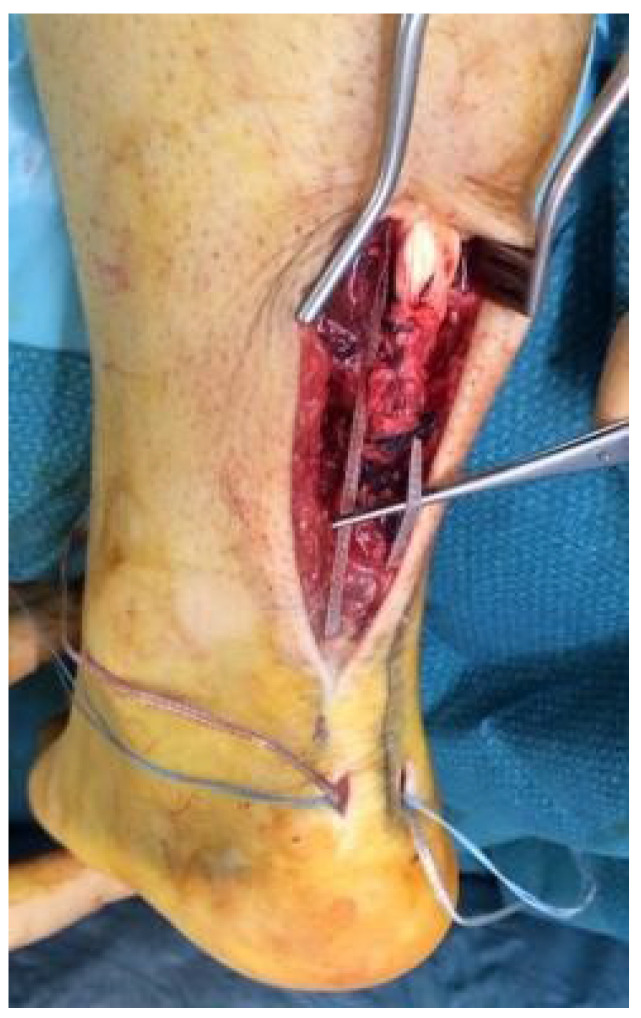
A repaired Achilles tendon with an internal brace in situ demonstrating FiberTape© while lax and when tensioned.

The tendon repair is then augmented with a 54″ (137 cm) 2 mm Arthrex FiberTape© mounted on a Mayo needle. With the ankle brought into dorsiflexion (to deliver more proximal tissue into the operative field), healthy non-injured tendon (or musculotendinous tissue) is harnessed by weaving it with the FiberTape© with 4 continuous locked sutures. The two free ends of the FiberTape© are then brought down alongside or through the distal tendon stump (Figure 1D). Two stab incisions are made medial and lateral to the midline on the posterior aspect of the calcaneum. The calcaneum docking ports are then drilled with an Arthrex SwiveLock 4.5 mm drill to a depth set by the skin protecting guide. The docking ports are tapped up to the laser line using the 4.75 mm SwiveLock tap. The two FiberTape© ends are tunnelled through the distal tendon tissue, under the skin, and are brought out through the two distal incisions (Figure 1E). The FiberTape© ends are secured in the standard way using two fully threaded knotless 4.75 mm bioabsorbable Arthrex SwiveLock anchors. It is essential that the FiberTape© tension is set at maximal ankle dorsiflexion, matching the contralateral ankle. By doing so, the FiberTape© augmentation is made to act as a “checkrein” to excessive dorsiflexion but without stress-shielding the repair site at lower degrees of dorsiflexion movement. This is akin to the “seatbelt” concept, with the tape lax during ankle plantarflexion and tightening as the ankle is brought into dorsiflexion. Figure 2 shows that the FiberTape© is lax at the “position of rest” for the ankle, with subsequent tightening up as the ankle is pushed into dorsiflexion.

**Figure 3:**
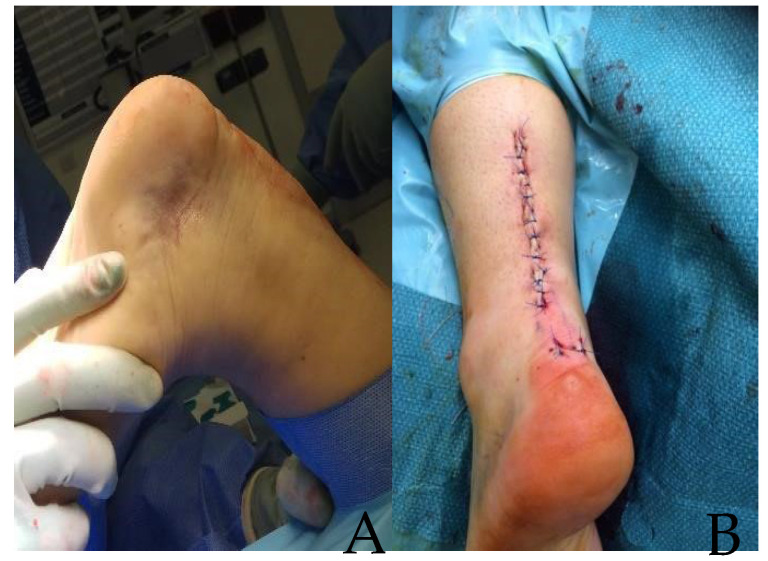
(**A**) The end construct of the Achilles tendon repair under maximal stress; (**B**) nylon closure.

Following repair, the strength of the construct should be tested by loading the ankle into full dorsiflexion (Figure 3A). The surgical wound is closed using interrupted non-absorbable sutures (3–0 nylon) into the skin (Figure 3B). The foot is then wrapped in wool and crepe bandages with no cast.

### 2.3. IBA-TR of the Quadriceps Tendon: Surgical Technique

**Figure 4:**
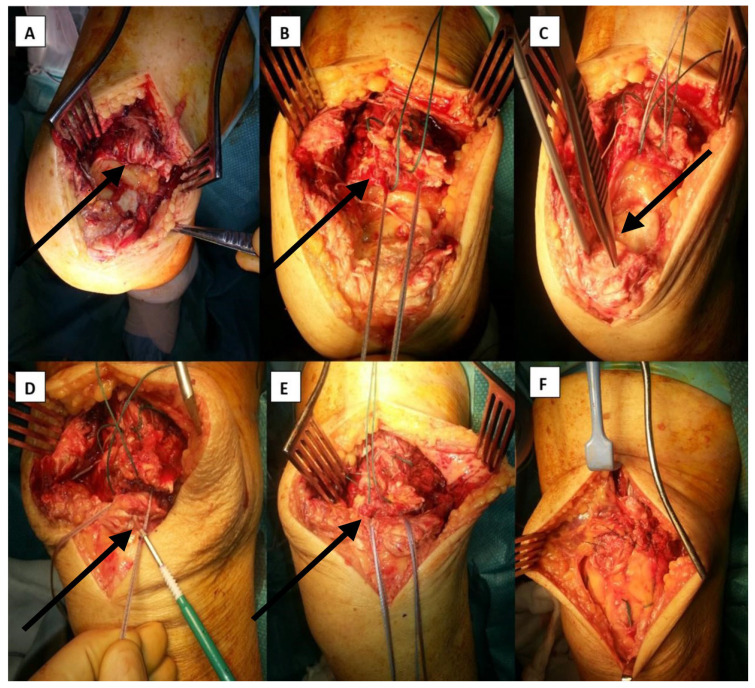
Step sequence for quadriceps tendon repair with internal brace implantation. The black arrows point directly to the step of procedure described with each letter. (**A**) ruptured quadriceps tendon; (**B**) Krackow quadriceps with both FiberTape© and polyester; (**C**) patella proximal aspect decorticated and drill holes for swivel lock anchors made; (**D**) tendon docked onto patella and tension set with swivel lock anchors; (**E**) showing reattached quadriceps tendon; (**F**) adjacent retinacular tissues and paratenon remnants sutured across repair site.

A midline longitudinal incision is made with full-thickness subcutaneous flaps. The injury is identified, and the avulsed tendon ends and the patella insertion are exposed. The haematoma is evacuated. Any adhesions and/or fibrotic material should be resected and the tendon ends debrided back to healthy edges, particularly in chronic tears. The proximal tendon is harnessed using a non-absorbable suture (2 polyester) or SutureTape in a Krackow configuration. If the tear is more proximal, and purely intratendinous, then a Krakow stitch can be raised on both the proximal and distal tendon stumps. This allows the tendon ends to be controlled and assists in reduction; the suture ends can later be used as part of the retinacular repair. A 54″ (137 cm) 2 mm Arthrex FiberTape© is then mounted on a Mayo needle and passed through the proximal tendon with 4 continuous locked (Krakow-type) sutures, and the two free ends of tape are brought down to the patella (Figure 4D). The proximal pole of the patella is then decorticated with a curette. The superior patellar bone is drilled twice with a SwiveLock 4.5 mm drill to a depth set by the skin protecting guide, with a gap of 1.5 to 2 cm between the drill holes. These are positioned in the 11 p.m. and 1 p.m. positions on the patellar “clockface”. The FiberTape© ends are then secured in the standard way using two fully threaded knotless 4.75 mm bioabsorbable Arthrex SwiveLock anchors, with the FiberTape© tension set to bring the proximal tendon closely up against the patellar bone (Figure 4E). In patients heavier than 120 kg, the authors suggest using a second FiberTape© weaved through another section of intact proximal quadriceps tendon, with the two additional SwiveLock docking points at the 2.30 pm and 9.30 pm positions on the patellar clockface.

The repair is then completed with a running retinacular suture using the free ends of the polyester sutures. The paratenon is repaired with a running 2–0 polyglactin 910 suture. In this type of repair, the augmentation using the FiberTape© and SwiveLocks acts similarly to a double suture anchor as well as an internal brace. The skin is closed using interrupted non-absorbable sutures (3–0 nylon). The leg is then wrapped in wool and crepe bandages with no cast.

### 2.4. IBA-TR of the Patellar Tendon: Surgical Technique

**Figure 5:**
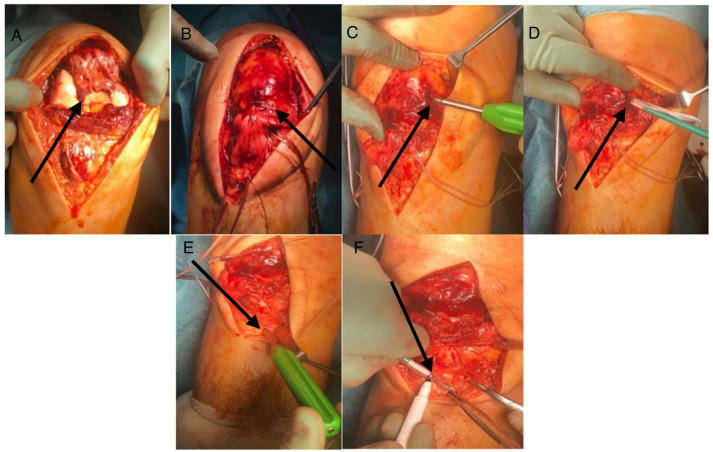
Step sequence for patellar tendon repair with internal brace implantation. The black arrows point directly to the step of procedure described with each letter. (**A**) mid-substance ruptured patellar tendon; (**B**) primary end-to-end repair of torn patellar tendon; (**C**) drilling and tapping patella at 3 pm position; (**D**) patella FiberTape© docked; (**E**) tibial tuberosity tapped; (**F**) FiberTape© secured to tibial tuberosity with SwiveLock anchor while knee is at 30 degrees of flexion.

**Figure 6:**
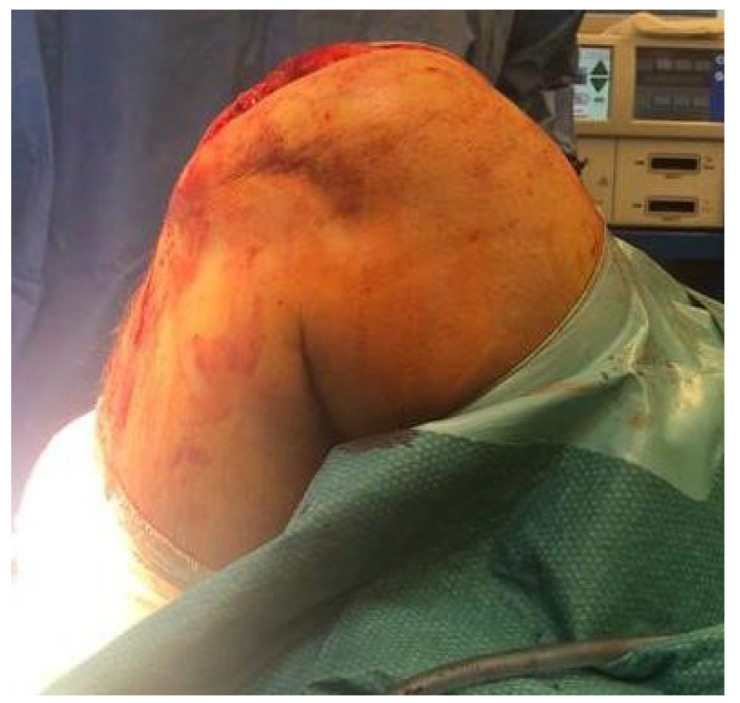
Assessing the strength of the reconstruction in deep flexion.

A midline longitudinal incision is made extending from the patella to the tibial tuberosity. The haematoma is evacuated and both the proximal and distal parts of the ruptured tendon are exposed (Figure 5A). A direct end-to-end tendon repair is performed utilising a Krakow configuration on both the proximal and distal ends of the tendon using 2 polyester or SutureTape. The leg is then brought into extension to allow apposition of the two ends of the repair; the four suture ends are paired up and 2 knots tied, taking care to match the patellar height and tendon length to the contralateral non-injured side. A continuous absorbable (2–0 polyglactin 910) epitendon suture is then weaved across the repair site to approximate the loose fragment ends and tendon strands and to smooth the contour of the tendon and complete the repair (Figure 5B). The paratenon fragments are sutured down across the top of the repair site (where possible) using absorbable suture (2–0 polyglactin 910). The tendon repair is then augmented with two sections of 54″ (137 cm) 2 mm Arthrex FiberTape©. The patellar bone is drilled with a SwiveLock 4.5 mm drill to a depth set by the skin protecting guide, at the 9 pm and 3 pm positions on the patellar clockface (Figure 5C). Two further drill holes are made at the level of the tibial tuberosity, approximately 1 cm medial and 1 cm lateral to the midline. Doubled-over FiberTape© ends are then secured in the standard way using two fully threaded knotless 4.75 mm bioabsorbable Arthrex SwiveLock anchors, first into the patellar sockets and then into the tibial tuberosity sockets, creating two double-stranded constructs. The FiberTape© tension is set to match the tension of the primary repair (but not tighter), again taking care to match the patellar height and tendon length of the contralateral non-injured side. In patients heavier than 120 kg, the authors have used 3 or even 4 strands of FiberTape© to bridge the gap between the tibia and the patella, with docking points at 3 pm, 5 pm, 7 pm and 9 pm on the patellar “clockface”. In this role, the FiberTape© augmentation acts as a “checkrein” to prevent proximal retraction of the patella or stretching of the patellar tendon, but without stress-shielding the actual repair site. Following the repair, the strength of the construct is tested by ranging the knee into deep flexion, assuring the surgeon that there is no failure at the repair site (Figure 6).

The skin is closed using interrupted non-absorbable sutures (3–0 nylon). The leg is then wrapped in wool and crepe bandages with no cast.

### 2.5. Post-Op Rehabilitation

**Figure 7:**
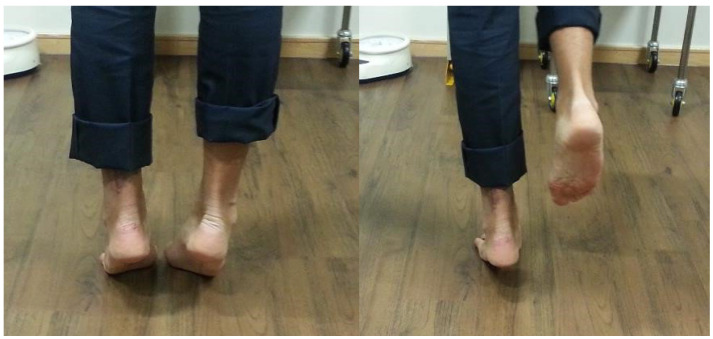
A patient at 6 weeks post-op performing concentric double- and single-stance heel raises.

Patients who undergo TA reconstruction are kept non-weightbearing in a walking boot (without wedges) for 2 weeks, and during that time they are given VTE prophylaxis. However, the foot and ankle are immediately mobilised, allowing an early range of motion out of the boot, and the patients are encouraged to mobilise their foot and ankle through “alphabet foot writing”, as well as performing gentle forefoot push-offs against an elastic Theraband as guided by their discomfort levels. The patients are then permitted to start weightbearing in the boot as tolerated at the 2-week post-operative mark, transitioning into trainers and/or barefoot walking from 4 weeks post-op. Concentric double-stance heel raises (Figure 7.), as well as the use of a static exercise bike or cross-trainer and swimming, are permitted from 6 weeks. Eccentric TA loading is permitted from 12 weeks. Light jogging is allowed from week 16.

Patients who undergo quadriceps or patellar tendon repair are put into a hinged knee brace for 6 weeks. They are allowed to fully bear weight on the operated leg, with the knee held in extension with the brace in situ. The knee is kept in extension full-time for the first 2 weeks to support wound healing, and straight leg raises are encouraged as well as some static quadriceps exercises. After 2 weeks, the knee brace is then fully unlocked to allow a free range of motion when seated, although the brace is locked in full knee extension when walking. After 6 weeks the brace is discarded and the patients are permitted to return to low-impact cyclical activities, including using a static bike or elliptical trainer, as well as swimming. A return to higher-impact activities is allowed from 12 weeks post-surgery, and running is allowed from 16 weeks.

## 3. Results

This single-surgeon cohort included 100 consecutive operated cases over the 8-year period from January 2014 to February 2022, followed up for a minimum of 6 months (Achilles tendon, 58 patients; quadriceps tendon, 25; patellar tendon, 17). Sixty-two patients were male, and thirty-eight were female. The mean age at the time of the surgery was 34 years (range 19–82 years). The mean time to mobilisation without a brace was 32 days. The average Tegner score at 12 months was 5.9 +/− 2.1.

At the 12-month follow up, no patient suffered from tendon repair failure. There were no recorded episodes of fracture or deep venous thrombosis. Three Achilles tendon patients (5.2%) developed superficial infections of their wounds, but none of them required a return to theatre, and they were successfully managed with oral antibiotics. Ninety-eight patients returned to their pre-operative levels of activity. Two quadriceps reconstruction patients (aged 62 and 77) did not return to the same levels of activity.

## 4. Discussion

Surgical repair is a mainstay treatment for quadriceps and patellar tendon repair, with conventional methods involving end-to-end suture repair. The reported rates of failure for this mode of fixation vary between 2% and 50%, with up to 10% of patients requiring revisions [16]. For conventional Achilles tendon repairs, the failure rate is reported to reach up to 5% and, furthermore, the return-to-sport rate is only 77.3% [17]. This article presents an alternative surgical technique that aims to allow for early patient mobilisation, which may further enhance their return to sports and reduce the rates of failure.

Several techniques related to augmenting tendons have been reported. These include the use of synthetic grafts, allografts and meshes to enhance biological healing, and repairs for larger tendon defects. However, allografts and mesh use can be associated with the introduction of bulky foreign material and increased risk of infection [18,19]. These techniques also carry the potential for donor site morbidity and are technically more demanding, with significant cost implications [19].

Rehabilitation from surgical tendon repair in the lower limb has traditionally involved immobilisation for an extended period of time [1]. A full range of motion is often not permitted until 3 months after the initial injury, which can result in significant stiffness and abnormal kinematics [8]. A recent systematic review found that most reported cases of quadriceps tendon rupture were immobilised in a cast for an average of 6 weeks in full extension. The same authors reported re-rupture rates of 2% amongst published studies, although overall muscle strength was reduced by 20–30% in comparison to the contralateral limb [20]. There is growing evidence supporting the importance of an early range of motion and protected physiological loading to optimise tendon healing and clinical outcomes [10].

However, early unprotected mobilisation with the current and widely employed techniques is associated with increased scar tissue and osteoclast activation, which can compromise the tendon–bone interface with a reduction in the trabecular architecture, hence necessitating protection of these constructs in the early post-operative period [21]. Indeed, Serino et al. assessed the adverse events associated with immobilisation post knee extensor non-internally braced tendon repair, comparing early versus delayed mobilisation. They identified an increased number of adverse events associated with an early range of motion and weightbearing, with the authors advocating a minimum of 6 weeks of fixed immobilisation [22]. A clear advantage of the techniques we have described lies with their ability to protect the constructs during the initial phases of healing, allowing a balance between allowing early mobilisation and protecting the tendon repair. The prevention of cast immobilisation also diminishes the potential joint fibrosis in combination with the promotion of reparative processes [1].

**Figure 8:**
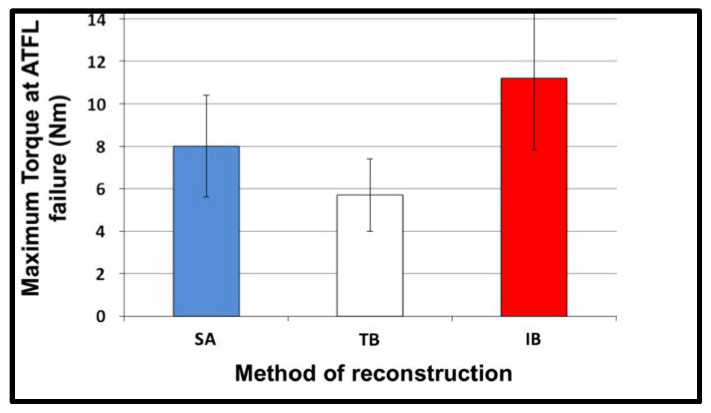
Comparing torque to failure for different methods of ATFL reconstruction: SA, suture anchor; TB, traditional Brostrom; IB, internal brace. Adapted from Schuh et al., 2016 [23].

Both biomechanical and clinical studies reflect improved outcomes with ligament repairs and reconstructions augmented with an internal brace [23,24,25,26,27]. These promising results have led to the increased use of internal bracing in ligament and tendon reconstruction [28,29]. Schuh et al. assessed the torque to failure of IBs and demonstrated a significant increase in torque to failure of the anterior talofibular ligament when reconstructed with an IB as compared to suture anchors (SAs) and a traditional Brostrom (TB) (Figure 8) [23].

Patients undergoing surgical fixation with internal bracing demonstrated significant improvements in their AOFAS scores two weeks post repair, as opposed to the non-braced control group who demonstrated no improvement [30].

The physiology of tendon repair is strongly related to its mechanical environment. In vitro studies analysing patellar tendon explants cultured in a medium of collagenase found an increase in both stiffness and elongation to failure for those specimens that had been pretensioned when compared to those in a zero-strain environment [31]. The cellular components of tendons are postulated to respond to their mechanical environments, with the extracellular matrix also modulated by various growth factors whose expression is increased with mechanical loading. More specifically, upstream transcription factors vital to limb and tendon formation are also thought to be expressed in response to cyclical mechanical stimulation [7]. In contrast, tendons under reduced mechanical stresses in animal models have been shown to display weaker biomechanical properties in comparison to those under tension [32].

A greater understanding of ligament and tendon healing has led to a renewed interest in developing techniques to augment the mechanical and biological environment in order to improve tendon healing and prevent morbidity. One such method is the use of internal bracing, which supports and protects loading of the tendon during its early healing and remodelling phases. Its role as a “seatbelt” or “checkrein” has been outlined with reference to the ankle, and its use has spread to repairs and reconstructions in the knee, wrist and elbow [33,34,35].

The “seatbelt” role of the internal brace allows for biological healing and revascularisation of ligaments [36,37,38,39]; however, its role in tendon reconstruction and repair remains underreported in the literature. In their cadaveric study of quadriceps tendon repairs, Rosseler et al. [40] found that suture tape improved the load distribution and withstood a greater load to ultimate failure. Schütte et al. published on improved outcomes with suture tape augmentation, although they employed a different technique to the one described by the senior author [41].

The technique we have described is not only of low technical complexity but also versatile, allowing it to adapt to the nature of the injury. The aforementioned quadriceps tendon repair utilised the FiberTape© anchor construct as part of the repair onto the patella. In more distal quadriceps avulsions, the anchor serves not only to reduce the tendon to the patella but also to prevent excessive loads across the primary repair; this facilitates early mobilisation and physiological loading whilst also reducing adverse events associated with enhanced rehabilitation and those of muscle atrophy related to immobilisation. The technique itself is of low technical difficulty and is reproducible with a short learning curve. It also represents a cost-effective method of augmenting tendon repair.

This case series represents a large cohort of tendon repairs, augmented with an internal brace, with no reported failures or adverse events. Our experience has reflected that this is a safe, reliable, and reproducible technique. It is noteworthy that this was a retrospective cohort study from a single surgeon, without a dedicated control group. Moreover, no formal post-operative imaging was arranged to assess the extent of tendon healing. Further studies with MR imaging at 12 months are recommended to fully elucidate the degree of healing and soft tissue reactions.

Early mobilisation also significantly reduced the incidence of VTE, which was zero for this case series. Moreover, 98 of the 100 patients returned to all their pre-injury activities. This compares favourably to a recent systematic review of patients receiving standard repairs of knee extensor tendon ruptures, which demonstrated that a third of patients did not return to their pre-injury levels of sporting activity [42]. In this study, in all cases we demonstrated a significant improvement in the return to pre-injury activity levels earlier than in patients treated with conventional techniques. This represents clinical concordance with laboratory data supporting early loading in optimising healing and reducing arthrofibrosis. The lead author of this paper endorses a more prominent role for the surgical management of tendon injuries where previous conservative measures were opted for in part due to the early facilitation of return to function and sporting activity.

## 5. Conclusions

This case series represents the largest cohort of tendon repairs augmented with an internal brace to date, with all the patients being allowed to mobilise early on after the procedure. Within the investigated timeframe, there were no reported failures or returns to theatre, indicating non-inferiority in comparison to conventional tendon repair techniques, although further comparative studies would be recommended. The technique described acts as a “checkrein” when the tendon is loaded in the early stages of healing, which, in turn, improves the long-term tendon strength. Overall, internal bracing in tendon repair has been demonstrated to be a cheap and widely available technique for improving patient outcomes in lower limb tendon repair, while remaining safe and low in complexity.

## 6. Limitations

This is foremost a report of a surgical technique with a limited cohort. Further evaluation of this technique is required with pre- and post-op validated scoring systems. Comparative studies between different modes of repair with a randomised control group comparing those with and without internal bracing are also indicated. This case series serves to advocate for the role of internal brace augmentation in relation to lower limb tendon injuries in both the acute and chronic settings. It needs to be noted that the low rate of failure must be interpreted with caution, considering that this was a single small-cohort study with a relatively short follow-up period. Possible complications associated with bony anchors within the patella, particularly in osteoporotic bone, need further research.

## Data Availability

The original contributions presented in this study are included in the article. Further inquiries can be directed to the corresponding author(s).
